# Daytime nap and nighttime sleep duration in relation to dyslipidemia among type 2 diabetes patients in eastern China: a cross-sectional study

**DOI:** 10.3389/fnut.2026.1861408

**Published:** 2026-06-30

**Authors:** Xiangyu Chen, Feng Lu, Jie Zhang, Qingfang He, Jieming Zhong

**Affiliations:** Department of Non-Communicable Disease Control and Prevention, Zhejiang Provincial Center for Disease Control and Prevention, Hangzhou, China

**Keywords:** dyslipidemia, lipids, nap, sleep duration, type 2 diabetes mellitus

## Abstract

**Objectives:**

To investigate associations of daytime nap and nighttime sleep duration with dyslipidemia among patients with type 2 diabetes (T2DM) in Eastern China.

**Methods:**

This investigation was conducted in Zhejiang Province, China, from March to November 2018. Multivariable logistic regression assessed associations of daytime nap and nighttime sleep duration with dyslipidemia. Restricted cubic spline (RCS) analyses using continuous sleep variables examined dose–response relationships. Sensitivity analyses (medication adjustment, reference category redefinition, modified Poisson regression) tested the robustness of the findings. Exploratory analyses evaluated individual lipid abnormalities. Odds ratios (ORs) and 95% confidence intervals (CIs) were estimated after confounder adjustment.

**Results:**

The study included 1,749 patients with T2DM, of whom 53.69% were classified as having dyslipidemia. In the fully adjusted logistic regression model, each standard deviation (SD) increment in daytime nap duration was significantly associated with 13% higher odds of dyslipidemia (OR = 1.13, 95% CI: 1.02–1.26). Compared with 1–30 min of daytime nap, >90 min was associated with 65% higher odds of dyslipidemia (OR = 1.65, 95% CI: 1.12–2.43). RCS analyses suggested a linear association between daytime nap duration and dyslipidemia (p for non-linearity > 0.05). For nighttime sleep duration, compared with >6–7 h, both <5 h and >8 h were associated with higher odds of dyslipidemia (OR = 2.16, 95% CI: 1.23–3.79 and OR = 1.50, 95% CI: 1.02–2.20, respectively). No significant association was observed for per-SD increment in nighttime sleep duration. RCS analyses demonstrated a significant U-shaped association between nighttime sleep duration and dyslipidemia. In exploratory analyses, prolonged daytime napping was associated with hypercholesterolemia, hypertriglyceridemia, and low HDL-C, whereas short nighttime sleep was primarily linked to hypertriglyceridemia and low HDL-C. Results from multiple sensitivity analyses were consistent with the primary findings, supporting the robustness of the observed associations.

**Conclusion:**

Among patients with T2DM, longer daytime nap duration and both short and long nighttime sleep duration were associated with higher odds of dyslipidemia, with distinct linear and U-shaped patterns. These sleep behaviors were associated with differences in specific lipid fractions, warranting further prospective investigation.

## 1 Introduction

Type 2 diabetes mellitus (T2DM) is a major public health concern worldwide and is characterized by chronic hyperglycemia accompanied by multiple metabolic abnormalities (1). Among these, dyslipidemia is highly prevalent and represents a key modifiable risk factor for both microvascular and macrovascular complications (2). Despite widespread use of lipid-lowering therapies, a considerable residual cardiovascular risk persists in patients with T2DM (3), highlighting the importance of identifying modifiable behavioral factors that may contribute to lipid abnormalities.

Sleep duration and timing are increasingly recognized as important determinants of metabolic regulation, including lipid homeostasis (4, 5). Experimental evidence suggests that disturbances in sleep may influence lipid metabolism through mechanisms such as circadian misalignment, neuroendocrine alterations, and increased sympathetic nervous system activity (6–10). Epidemiological studies have also reported associations between abnormal sleep duration and adverse lipid profiles, including elevated triglycerides and reduced high-density lipoprotein cholesterol (4, 11–13).

Daytime nap and nighttime sleep duration represent two distinct but interrelated aspects of sleep behavior (14). While short daytime naps may have restorative effects, prolonged daytime nap has been linked to adverse metabolic outcomes, including obesity and insulin resistance (15). Similarly, both short and long nighttime sleep durations have been associated with increased cardiometabolic risk, with several studies suggesting a U-shaped relationship with dyslipidemia (16). However, some results of prior studies remain inconsistent, with some studies reporting positive associations, whereas others have reported null or inverse findings (17). Moreover, most existing studies have been conducted in general populations, with limited focus on patients with T2DM.

Given the presence of insulin resistance, altered lipid metabolism, and elevated baseline cardiometabolic risk in T2DM, sleep-related metabolic disturbances may have distinct or amplified effects in this population (18, 19). In addition, daytime napping is common in Chinese populations (20), and sleep patterns may differ from those observed in western settings. However, evidence regarding the dose–response associations of daytime nap and nighttime sleep duration with dyslipidemia in patients with T2DM, particularly in Eastern China, remains limited.

Therefore, this study aimed to investigate the associations and potential dose–response relationships of daytime nap duration and nighttime sleep duration with dyslipidemia among patients with T2DM in Eastern China. The findings may contribute to a better understanding of the relationship between sleep behaviors and dyslipidemia in this high-risk population.

## 2 Materials and methods

### 2.1 Subjects

**Figure 1:**
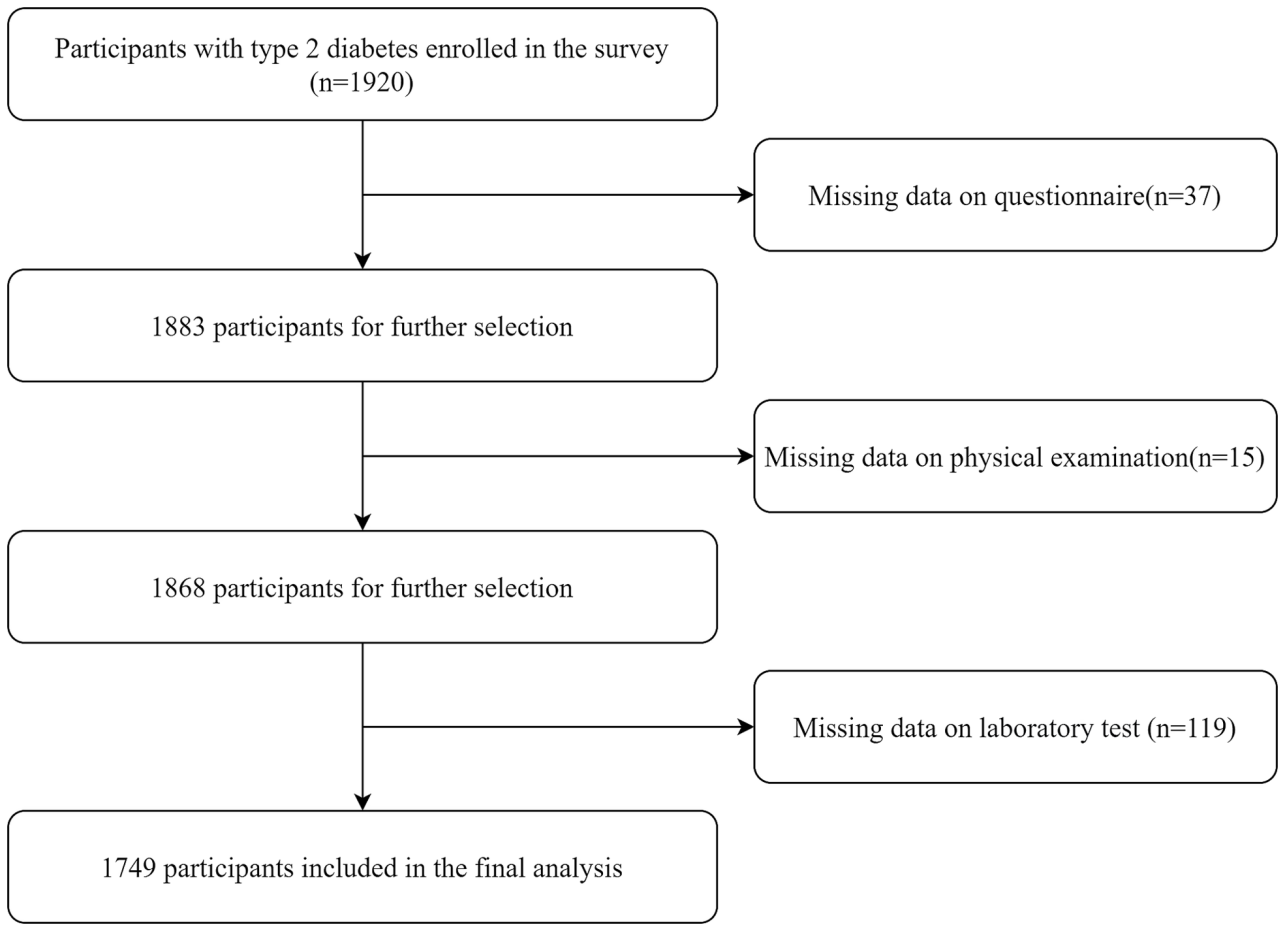
Flowchart of the participant selection process.

Participants in this study were recruited from the Zhejiang Diabetic Complications Study, a subcomponent of the China National Diabetic Complications Study. The investigation was carried out from March to November 2018 and targeted T2DM patients aged ≥18 years who were registered local residents, with T2DM diagnosis based on physician diagnosis and medical records from local healthcare institutions. A multi-stage random sampling strategy was applied to identify eligible participants. Initially, two counties and two districts were randomly selected from the province. Subsequently, four townships or subdistricts were randomly chosen within each selected county and district. In the final stage, 120 individuals with a diagnosis of T2DM were randomly sampled, with stratification to maintain balance across sex and age categories. This process resulted in a total sample size of 1,920 participants. Further details regarding the study design and methods are available in the published protocol (21). After excluding individuals with missing data, 1,749 participants were included in the final analysis. The participant selection process is illustrated in Figure 1.

### 2.2 Data collection

Data on demographic characteristics, lifestyle behaviors, and medical history were collected through standardized face-to-face interviews conducted by trained staff from local centers for disease control and prevention and primary healthcare institutions. Anthropometric measurements, including height and weight, were obtained using standardized procedures, and body mass index (BMI) was calculated as weight (kg) divided by height squared (m^2^). Blood pressure was measured using a calibrated electronic sphygmomanometer after at least 5 min of rest. Fasting venous blood samples were collected after an overnight fast of at least 8 h. Biochemical parameters, including fasting plasma glucose (FPG), glycated hemoglobin (HbA1c), total cholesterol (TC), triglycerides (TG), low-density lipoprotein cholesterol (LDL-C), and high-density lipoprotein cholesterol (HDL-C), were measured using standardized laboratory methods. Serum lipid levels were determined using enzymatic assays with a Roche cobas c701 automated analyzer (Roche, Switzerland). FPG was measured using the hexokinase method, and HbA1c was assessed using high-performance liquid chromatography (Bio-Rad D10 Hemoglobin Analyzer, United States). Daytime nap duration and nighttime sleep duration were self-reported using structured questionnaires.

### 2.3 Definition of variables

Dyslipidemia was defined according to the 2016 Chinese guidelines for the management of dyslipidemia in adults as the presence of any of the following individual lipid abnormalities: hypercholesterolemia (total cholesterol [TC] ≥ 6.2 mmol/L), hypertriglyceridemia (triglycerides [TG] ≥ 2.3 mmol/L), elevated low-density lipoprotein cholesterol (LDL-C ≥ 4.1 mmol/L), low high-density lipoprotein cholesterol (HDL-C < 1.0 mmol/L), or a self-reported prior diagnosis of dyslipidemia (22). Hypertension was defined as systolic blood pressure ≥140 mmHg and/or diastolic blood pressure ≥90 mmHg, or a self-reported history of hypertension (23). Educational attainment was categorized as secondary school or below, senior high school or above. Residential status was classified as urban or rural. Current smoking was defined as daily or occasional cigarette use, and alcohol consumption was defined as any alcohol intake within the preceding 30 days. Regular exercise was defined as engaging in moderate-intensity or higher physical activity on at least 5 days per week, with a duration of at least 30 min per session (24). Family history of dyslipidemia was assessed based on self-report. Abnormal FPG and HbA1c levels were defined as ≥7.0 mmol/L and ≥7.0%, respectively. Daytime nap duration was assessed using the following question: “How many minutes do you usually nap, or sleep at any fixed time during the daytime, within a typical day?” Based on previous literature regarding daytime napping and health outcomes (25), as well as the distribution of nap duration in the study population, daytime nap duration was categorized into five groups: 0 min, 1–30 min, 31–60 min, 61–90 min, and >90 min. Nighttime sleep duration was assessed using the following question: “How many hours do you usually sleep at night on a typical day?” Based on previous literature on sleep duration and metabolic health (26), together with the distribution of sleep duration in the study population, nighttime sleep duration was categorized into five groups: <5 h, 5–6 h, >6–7 h, >7–8 h, and >8 h.

### 2.4 Statistical analysis

Continuous variables were summarized as mean (standard deviation [SD]) or median (interquartile range [IQR]) according to their distribution, while categorical variables were presented as counts and percentages. For comparisons between two groups, Student’s *t*-test or the Mann–Whitney U test was used for continuous variables, as appropriate, and the chi-square test was used for categorical variables. For comparisons across multiple groups, one-way analysis of variance (ANOVA) or the Kruskal–Wallis test was used for continuous variables, as appropriate, and the chi-square test was used for categorical variables. When the overall ANOVA result was statistically significant, post-hoc pairwise comparisons were performed using Tukey’s Honestly Significant Difference (HSD) test. When the overall Kruskal–Wallis test result was statistically significant, post-hoc pairwise comparisons were performed using Dunn’s test with Bonferroni correction. For categorical variables, post-hoc pairwise comparisons were additionally conducted using the chi-square test or Fisher’s exact test, as appropriate, and Bonferroni correction was applied for multiple comparisons. Multivariable logistic regression models were constructed to evaluate the associations between daytime nap duration, nighttime sleep duration, and dyslipidemia. Odds ratios (ORs) and 95% confidence intervals (CIs) were estimated. Potential multicollinearity between daytime nap duration and nighttime sleep duration was evaluated using variance inflation factors (VIFs), and all VIF values were below 2, indicating no substantial multicollinearity. Restricted cubic spline (RCS) functions were fitted within logistic regression models using continuous daytime nap duration and nighttime sleep duration variables to examine potential dose–response relationships with dyslipidemia. Three knots were placed at the 10th, 50th, and 90th percentiles of each sleep variable distribution. The reference values for OR estimation were set at 30 min for daytime nap duration and 7 h for nighttime sleep duration, with OR = 1 representing these reference exposure values in the spline curves. Based on the RCS results and previous epidemiological studies reporting these ranges as relatively low-risk or recommended sleep durations, daytime nap duration and nighttime sleep duration were further analyzed as categorical variables. The reference categories were 1–30 min for daytime nap duration and >6–7 h for nighttime sleep duration (26, 27). Additional analyses using per-SD increase were also conducted for both daytime nap duration and nighttime sleep duration to assess the robustness of the findings. Three sequential models were specified: Model 1 (crude model), with no covariates included; Model 2, adjusted for age and sex; and Model 3 (fully adjusted model), further adjusted for educational attainment, residence, BMI, elevated HbA1c, hypertension, smoking, drinking, regular exercise, duration of diabetes, family history of dyslipidemia, daytime nap duration, and nighttime sleep duration. When examining daytime nap duration, nighttime sleep duration was included as a covariate, and vice versa. Three sensitivity analyses were conducted to evaluate the robustness of the findings. First, additional adjustment was performed for lipid-lowering therapy, glucose-lowering therapy, insulin use and antihypertensive medication beyond the covariates included in the fully adjusted model. Second, to assess the influence of reference category selection, participants with no daytime nap (0 min) and those with very short naps (1–30 min) were combined into a single reference group, which was compared with longer nap duration categories. Third, because dyslipidemia was highly prevalent in the study population, modified Poisson regression model was used to estimate prevalence ratios (PRs) and 95% CIs. The same covariates as those included in the fully adjusted logistic regression model were applied. Furthermore, as exploratory analyses, we evaluated the associations between sleep behaviors (daytime napping and nighttime sleep duration) and individual lipid abnormalities, specifically hypercholesterolemia, hypertriglyceridemia, elevated LDL-C and low HDL-C. Multivariable logistic regression models, applying the same covariate adjustments as in Model 3, were utilized to estimate ORs and 95% CIs for each specific lipid component. All statistical analyses were conducted using R software (version 4.2.1; R Foundation for Statistical Computing). A two-sided *p*-value <0.05 was considered statistically significant.

## 3 Results

### 3.1 General characteristics of the participants

A total of 1,749 patients with T2DM were included, among whom 939 (53.7%) were classified as having dyslipidemia. Of these, 532 (56.66%) were identified based on laboratory measurements alone, 147 (15.65%) based on self-reported history only, and 260 (27.69%) met both criteria. The mean age of the total participants was 57.21 ± 10.16 years, and no significant difference was observed between groups. Compared to the non-dyslipidemia group, participants with dyslipidemia were more likely to be male and urban residents (both *p* < 0.05). The dyslipidemia group exhibited significantly higher body mass index (BMI), systolic and diastolic blood pressure, as well as poorer glycemic control indicated by higher FPG and HbA1c levels (all *p* < 0.05). Notably, while marital status, nighttime sleep duration, alcohol consumption, regular exercise habits, diabetes duration, and LDL-C levels did not differ significantly between the two groups (all *p* > 0.05), the median daytime nap duration was significantly longer in the dyslipidemia group (*p* < 0.05). Additionally, the prevalence of hypertension, smoking, and family history of dyslipidemia was significantly higher in the dyslipidemia group (all *p* < 0.05). Detailed results are presented in Table 1.

**Table 1:** Basic characteristics of the subjects stratified by dyslipidemia status (*n* = 1,749).

Characteristics	Total (*n* = 1,749)	Subjects without dyslipidemia (*n* = 810)	Subjects with dyslipidemia (*n* = 939)	*t*/χ^2^/*z*	*p*-value
Age (years), [means ± SD]	57.21 ± 10.16	57.57 ± 10.18	56.91 ± 10.13	1.35^a^	0.177
Sex, *n* (%)				6.14^b^	0.013
Male	872 (49.86)	378 (46.67)	494 (52.61)		
Female	877 (50.14)	432 (53.33)	445 (47.39)		
Educational level, *n* (%)				7.82^b^	0.005
Secondary school and lower	1,535 (87.76)	730 (90.12)	805 (85.73)		
Senior high school or above	214 (12.24)	80 (9.88)	134 (14.27)		
Marriage status, *n* (%)				0.06^b^	0.801
Married	1,599 (91.42)	742 (91.60)	857 (91.27)		
Others	150 (8.58)	68 (8.40)	82 (8.73)		
Residence, *n* (%)				22.29^b^	<0.001
Rural	874 (49.97)	454 (56.05)	420 (44.73)		
Urban	875 (50.03)	356 (43.95)	519 (55.27)		
BMI (kg/m^2^), [means ± SD]	24.77 ± 3.44	23.86 ± 3.22	25.55 ± 3.43	−10.57^a^	<0.001
SBP (mmHg), [means ± SD]	134.16 ± 17.55	132.26 ± 17.34	135.80 ± 17.57	−4.22^a^	<0.001
DBP (mmHg), [means ± SD]	77.42 ± 10.08	75.87 ± 9.76	78.77 ± 10.17	−6.06^a^	<0.001
Nighttime sleep duration (hours), [means ± SD]	6.90 ± 1.33	6.93 ± 1.25	6.88 ± 1.38	0.74^a^	0.460
Daytime nap duration (minutes), [median (IQR)]	30.00 (0.00, 60.00)	30.00 (0.00, 60.00)	45.00 (0.00, 60.00)	−4.00^c^	<0.001
TG (mmol/L), [median (IQR)]	1.60 (1.12, 2.42)	1.21 (0.94, 1.57)	2.32 (1.55, 3.30)	−24.23^c^	<0.001
TC (mmol/L), [means±SD]	4.66 ± 1.07	4.47 ± 0.72	4.82 ± 1.28	−7.33^a^	<0.001
HDL-C (mmol/L), [means ± SD]	1.25 ± 0.36	1.44 ± 0.30	1.09 ± 0.32	23.27^a^	<0.001
LDL-C (mmol/L), [means ± SD]	2.73 ± 0.90	2.74 ± 0.67	2.73 ± 1.06	0.35^a^	0.727
FPG (mmol/L), [means ± SD]	7.94 ± 2.57	7.74 ± 2.41	8.11 ± 2.70	−3.04^a^	0.002
HbA1c(%), [means ± SD]	7.27 ± 1.49	7.16 ± 1.48	7.37 ± 1.48	−2.96^a^	0.003
Hypertension, *n* (%)	1,096 (62.66)	437 (53.95)	659 (70.18)	48.96^b^	<0.001
Smoking, *n* (%)	434 (24.81)	170 (20.99)	264 (28.12)	11.84^b^	<0.001
Drinking, *n* (%)	644 (36.82)	285 (35.19)	359 (38.23)	1.74^b^	0.188
Regular exercise, *n* (%)	289 (16.52)	121 (14.94)	168 (17.89)	2.75^b^	0.097
Duration of diabetes (years), [median (IQR)]	6.00 (3.00, 10.00)	6.00 (3.00, 10.00)	5.00 (3.00, 10.00)	−1.68^c^	0.092
Family history of dyslipidemia, *n* (%)	260 (14.87)	77 (9.51)	183 (19.49)	34.24^b^	<0.001

a Student’s t-test.

b Chi-square test.

c Mann-Whitney U test.

BMI, body mass index; SBP, systolic blood pressure; DBP, diastolic blood pressure; TG, triglycerides; TC, total cholesterol; HDL-C, high density lipoprotein-cholesterol; LDL-C, low density lipoprotein-cholesterol; FPG, fasting plasma glucose; HbA1c, hemoglobin A1c.

### 3.2 Basic characteristics of the subjects according to daytime nap duration

Participants were categorized into five groups based on daytime nap duration (0, 1–30, 31–60, 61–90, and >90 min). As presented in Table 2, significant overall differences were observed across groups in age, educational level, residence, BMI, and several health-related factors (all overall *p* < 0.05). The prevalence of dyslipidemia, elevated HbA1c and hypertension varied significantly across nap duration categories (both overall *p* < 0.05). Lifestyle behaviors, including smoking, alcohol consumption, and regular exercise, also showed significant overall differences among groups (all overall *p* < 0.05). In contrast, sex distribution, marital status, elevated FPG, and family history of dyslipidemia were comparable across groups. In post-hoc pairwise comparisons, the proportion of urban residents was significantly lower in the 0-min nap group than in the other nap duration groups. No other pairwise comparisons reached statistical significance.

**Table 2:** Basic characteristics of the subjects according to daytime nap duration, minutes (*n* = 1,749).

Characteristics	Total (*n* = 1,749)	0 (*n* = 643)	1-30 (*n* = 269)	31-60 (*n* = 476)	61-90 (*n* = 133)	>90 (*n* = 228)	Overall *p*
Age (years), [means ± SD]	57.21 ± 10.16	55.80 ± 10.12	56.67 ± 9.75	58.56 ± 10.04	60.42 ± 10.44	57.15 ± 10.12	<0.001
Sex, *n* (%)							0.068
Male	872 (49.86)	304 (47.28)	121 (44.98)	255 (53.57)	73 (54.89)	119 (52.19)	
Female	877 (50.14)	339 (52.72)	148 (55.02)	221 (46.43)	60 (45.11)	109 (47.81)	
Educational level, *n* (%)							<0.001
Secondary school and lower	1,535 (87.76)	582 (90.51)	221 (82.16)	401 (84.24)	119 (89.47)	212 (92.98)	
Senior high school or above	214 (12.24)	61 (9.49)	48 (17.84)	75 (15.76)	14 (10.53)	16 (7.02)	
Marriage status, *n* (%)							0.056
Married	1,599 (91.42)	594 (92.38)	242 (89.96)	445 (93.49)	117 (87.97)	201 (88.16)	
Others	150 (8.58)	49 (7.62)	27 (10.04)	31 (6.51)	16 (12.03)	27 (11.84)	
Residence, *n* (%)							<0.001
Rural	874 (49.97)	416 (64.70)	116 (43.12)	219 (46.01)	48 (36.09)	75 (32.89)	
Urban	875 (50.03)	227 (35.30)	153 (56.88)	257 (53.99)	85 (63.91)	153 (67.11)	
BMI (kg/m^2^), [means ± SD]	24.77 ± 3.44	24.82 ± 3.47	24.26 ± 3.26	24.81 ± 3.37	24.25 ± 3.43	25.42 ± 3.57	0.002
Dyslipidemia, *n* (%)	939 (53.69)	318 (49.46)	132 (49.07)	267 (56.09)	72 (54.14)	150 (65.79)	<0.001
Elevated FPG, *n* (%)	985 (56.32)	342 (53.19)	159 (59.11)	271 (56.93)	79 (59.40)	134 (58.77)	0.336
Elevated HbA1c, *n* (%)	854 (48.83)	292 (45.41)	123 (45.72)	237 (49.79)	74 (55.64)	128 (56.14)	0.021
Hypertension, *n* (%)	1,096 (62.66)	376 (58.48)	152 (56.51)	313 (65.76)	95 (71.43)	160 (70.18)	<0.001
Smoking, *n* (%)	434 (24.81)	166 (25.82)	48 (17.84)	120 (25.21)	33 (24.81)	67 (29.39)	0.041
Drinking, *n* (%)	644 (36.82)	206 (32.04)	106 (39.41)	192 (40.34)	40 (30.08)	100 (43.86)	0.002
Regular exercise, *n* (%)	289 (16.52)	79 (12.29)	63 (23.42)	86 (18.07)	28 (21.05)	33 (14.47)	<0.001
Duration of diabetes (years), [median (IQR)]	6.00 (3.00, 10.00)	5.00 (3.00, 10.00)	6.00 (4.00, 11.00)	6.00 (3.00, 10.00)	8.00 (4.00, 15.00)	6.00 (3.00, 10.00)	0.002
Family history of dyslipidemia, *n* (%)	260 (14.87)	80 (12.44)	39 (14.50)	87 (18.28)	24 (18.05)	30 (13.16)	0.062

BMI, body mass index; FPG, fasting plasma glucose; HbA1c, hemoglobin A1c.

### 3.3 Basic characteristics of the subjects according to nighttime sleep duration

Participants were categorized into five groups based on nighttime sleep duration (<5, 5–6, >6–7, >7–8, and >8 h). As presented in Table 3, significant overall differences were observed across groups in age, residence, BMI, dyslipidemia, and alcohol consumption (all overall *p* < 0.05). In contrast, no significant differences were found in sex, educational level, marital status, elevated FPG, HbA1c, hypertension, smoking, regular exercise, duration of diabetes, or family history of dyslipidemia across the five groups. In post-hoc pairwise comparisons, no statistically significant differences were observed between specific sleep duration groups for the variables with significant overall differences.

**Table 3:** Basic characteristics of the subjects according to nighttime sleep duration, hours (*n* = 1,749).

Characteristics	Total (*n* = 1,749)	<5 (*n* = 71)	5, 6 (*n* = 524)	>6, 7 (*n* = 488)	>7, 8 (*n* = 504)	>8 (*n* = 162)	Overall *p*
Age (years), [means ± SD]	57.21 ± 10.16	60.84 ± 7.15	58.32 ± 9.45	56.31 ± 10.13	56.28 ± 10.89	57.67 ± 10.55	<0.001
Sex, *n* (%)							0.087
Male	872 (49.86)	33 (46.48)	273 (52.10)	237 (48.57)	235 (46.63)	94 (58.02)	
Female	877 (50.14)	38 (53.52)	251 (47.90)	251 (51.43)	269 (53.37)	68 (41.98)	
Educational level, *n* (%)							0.127
Secondary school and lower	1,535 (87.76)	67 (94.37)	466 (88.93)	421 (86.27)	434 (86.11)	147 (90.74)	
Senior high school or above	214 (12.24)	4 (5.63)	58 (11.07)	67 (13.73)	70 (13.89)	15 (9.26)	
Marriage status, *n* (%)							0.781
Married	1,599 (91.42)	65 (91.55)	483 (92.18)	449 (92.01)	454 (90.08)	148 (91.36)	
Others	150 (8.58)	6 (8.45)	41 (7.82)	39 (7.99)	50 (9.92)	14 (8.64)	
Residence, *n* (%)							0.015
Rural	874 (49.97)	29 (40.85)	238 (45.42)	246 (50.41)	268 (53.17)	93 (57.41)	
Urban	875 (50.03)	42 (59.15)	286 (54.58)	242 (49.59)	236 (46.83)	69 (42.59)	
BMI (kg/m^2^), [means ± SD]	24.77 ± 3.44	24.81 ± 3.02	24.94 ± 3.28	24.67 ± 3.25	24.48 ± 3.51	25.34 ± 4.26	0.045
Dyslipidemia, *n* (%)	939 (53.69)	48 (67.61)	285 (54.39)	245 (50.20)	264 (52.38)	97 (59.88)	0.028
Elevated FPG, *n* (%)	985 (56.32)	42 (59.15)	303 (57.82)	267 (54.71)	282 (55.95)	91 (56.17)	0.869
Elevated HbA1c, *n* (%)	854 (48.83)	34 (47.89)	268 (51.15)	231 (47.34)	245 (48.61)	76 (46.91)	0.766
Hypertension, *n* (%)	1,096 (62.66)	49 (69.01)	347 (66.22)	287 (58.81)	309 (61.31)	104 (64.20)	0.103
Smoking, *n* (%)	434 (24.81)	18 (25.35)	144 (27.48)	116 (23.77)	106 (21.03)	50 (30.86)	0.053
Drinking, *n* (%)	644 (36.82)	29 (40.85)	219 (41.79)	176 (36.07)	156 (30.95)	64 (39.51)	0.007
Regular exercise, *n* (%)	289 (16.52)	9 (12.68)	82 (15.65)	88 (18.03)	87 (17.26)	23 (14.20)	0.610
Duration of diabetes (years), [median (IQR)]	6.00 (3.00, 10.00)	6.00 (3.00, 11.00)	6.00 (3.00, 10.00)	6.00 (3.00, 10.00)	6.00 (3.00, 10.00)	6.00 (3.00, 12.00)	0.818
Family history of dyslipidemia, *n* (%)	260 (14.87)	9 (12.68)	92 (17.56)	69 (14.14)	72 (14.29)	18 (11.11)	0.248

BMI, body mass index; FPG, fasting plasma glucose; HbA1c, hemoglobin A1c.

### 3.4 Dose–response analysis of daytime nap duration in relation to dyslipidemia

**Figure 2:**
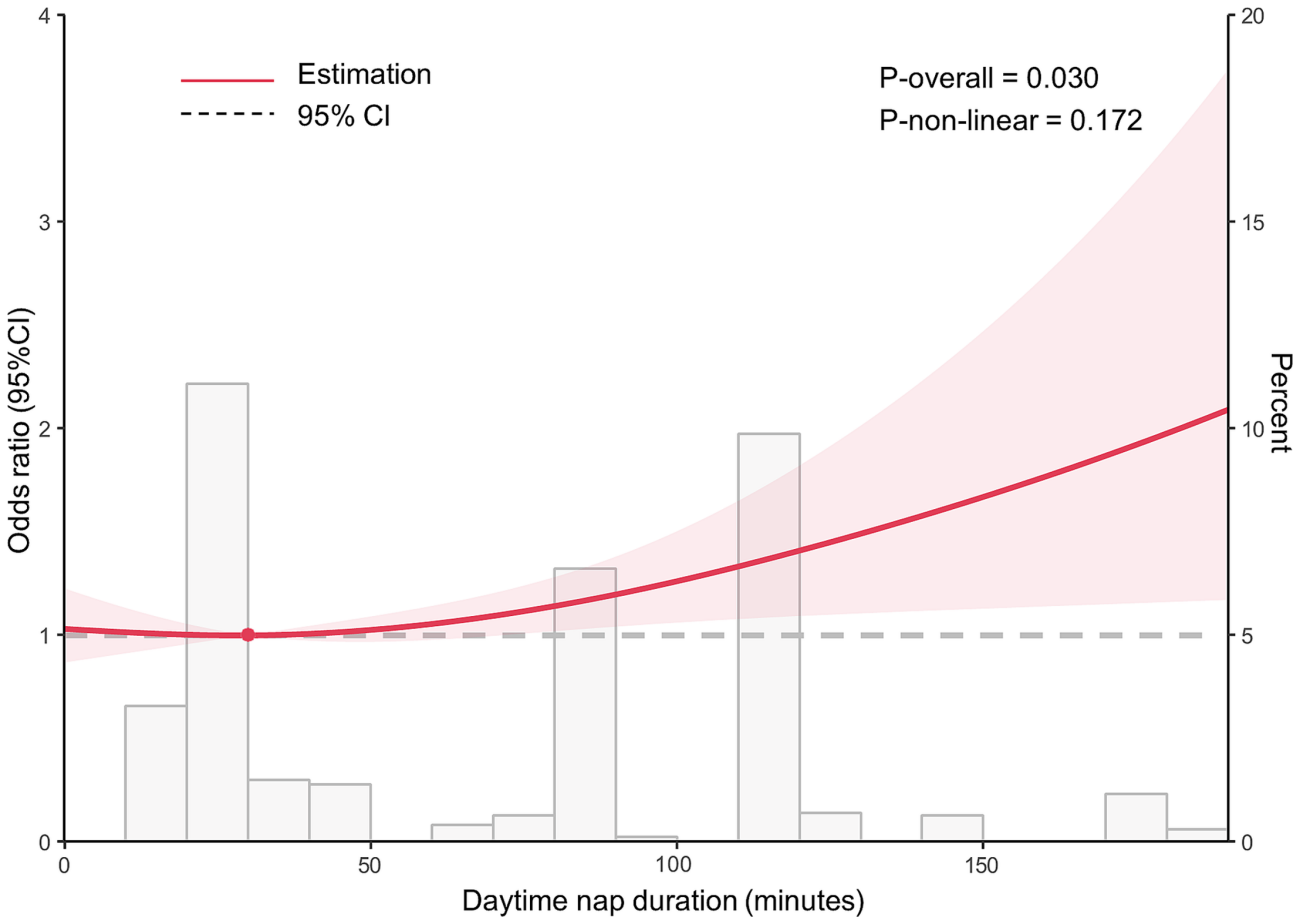
Dose–response association between daytime nap duration and dyslipidemia. The figure shows the association between daytime nap duration and the odds of dyslipidemia, with 95% confidence intervals (CIs), estimated using a restricted cubic spline model. The x-axis represents daytime nap duration, and the y-axis represents odds ratios (ORs) for dyslipidemia. The solid line indicates the estimated ORs, and the shaded area represents the corresponding 95% CIs. Analyses were adjusted for age, sex, educational attainment, residence, body mass index (BMI), nighttime sleep duration, elevated HbA1c, hypertension, smoking status, alcohol consumption, regular exercise, duration of diabetes, and family history of dyslipidemia.

The dose–response relationship between daytime nap duration and dyslipidemia was assessed using a RCS model, with 30 min of daytime nap set as the reference value (OR = 1). As shown in Figure 2, no significant non-linear association was observed after adjusting for the covariates included in Model 3 (*p* for non-linearity >0.05). The result indicated a linear positive association between daytime nap duration and the odds of dyslipidemia, with the odds gradually increasing as nap duration exceeded the 30-min reference.

### 3.5 Dose–response analysis of nighttime sleep duration in relation to dyslipidemia

**Figure 3:**
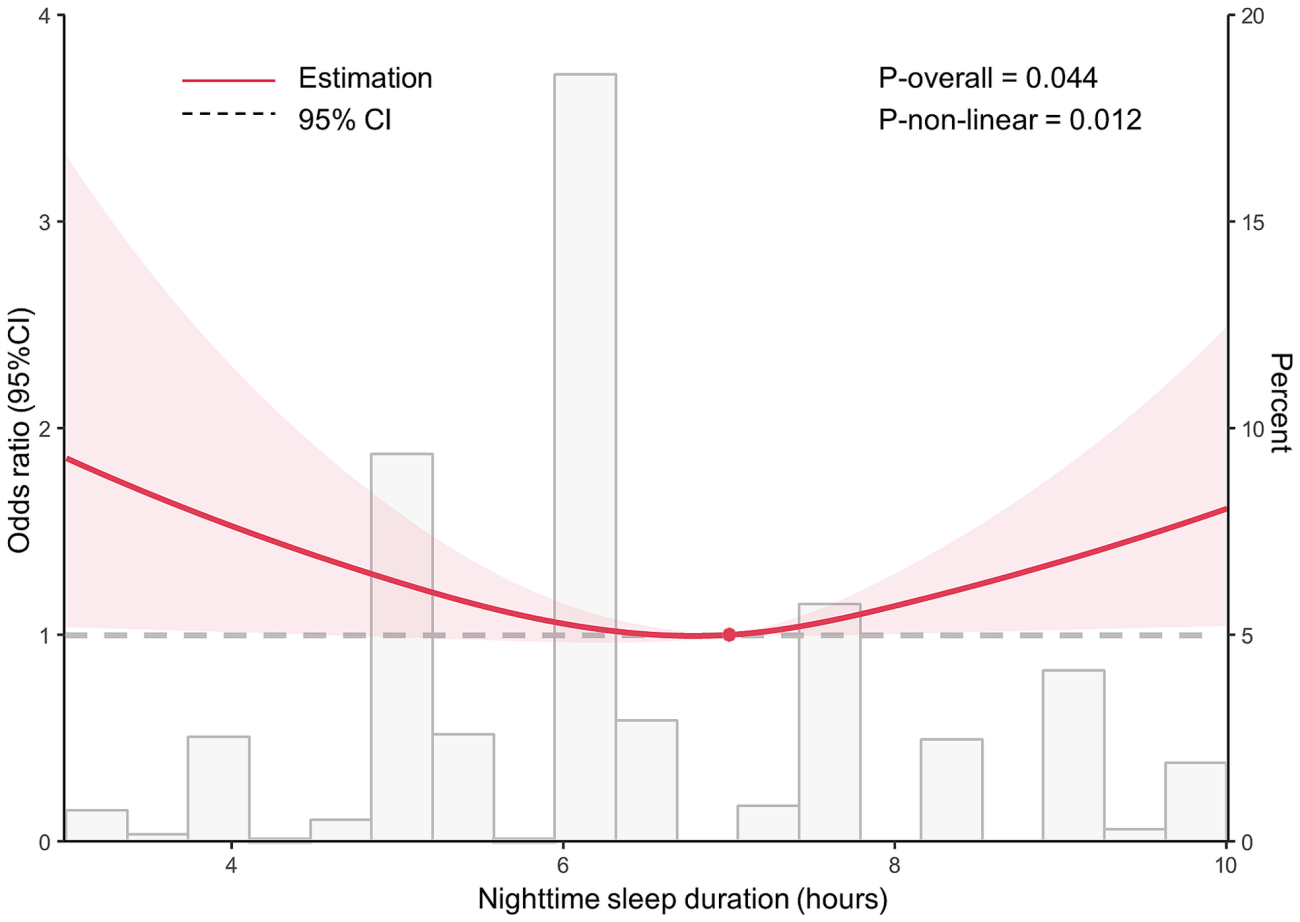
Dose–response association between nighttime sleep duration and dyslipidemia. The figure shows the association between nighttime sleep duration and the odds of dyslipidemia, with 95% confidence intervals (CIs), estimated using a restricted cubic spline model. The x-axis represents nighttime sleep duration, and the y-axis represents odds ratios (ORs) for dyslipidemia. The solid line indicates the estimated ORs, and the shaded area represents the corresponding 95% CIs. Analyses were adjusted for age, sex, educational attainment, residence, body mass index (BMI), daytime nap duration, elevated HbA1c, hypertension, smoking status, alcohol consumption, regular exercise, duration of diabetes, and family history of dyslipidemia.

The dose–response relationship between nighttime sleep duration and dyslipidemia was evaluated using a RCS model, with 7 h of nighttime sleep set as the reference value (OR = 1). As shown in Figure 3, a significant non-linear association was observed after adjusting for the covariates included in Model 3 (*p* for non-linearity <0.05). The result indicated a U-shaped relationship between nighttime sleep duration and the odds of dyslipidemia, with the lowest odds observed at 7 h of sleep. Both shorter and longer sleep durations were associated with progressively higher odds of dyslipidemia compared with the reference value.

### 3.6 Association of daytime nap and nighttime sleep duration with dyslipidemia

Table 4 presents the associations of daytime nap duration and nighttime sleep duration with dyslipidemia across three models. In the fully adjusted model (Model 3), each SD increment in nap duration was significantly associated with higher odds of dyslipidemia (OR = 1.13, 95% CI: 1.02–1.26). Additionally, nap duration >90 min was significantly associated with higher odds of dyslipidemia compared with the reference group (1–30 min) (OR = 1.65, 95% CI: 1.12–2.43), whereas other nap categories showed no significant associations. For nighttime sleep duration, in Model 3, both short (<5 h) and long (>8 h) sleep durations were significantly associated with higher odds of dyslipidemia compared with the reference group (>6–7 h) (OR = 2.16, 95% CI: 1.23–3.79; OR = 1.50, 95% CI: 1.02–2.20, respectively). No significant associations were found for 5–6 h or >7–8 h of sleep, nor for per SD increment in nighttime sleep duration (OR = 1.01, 95% CI: 0.91–1.11).

**Table 4:** Association of daytime nap and nighttime sleep duration with dyslipidemia (*n* = 1,749).

Characteristics	Model1		Model2		Model3	
	OR (95% CI)	*p*	OR (95% CI)	*p*	OR (95% CI)	*p*
Daytime nap duration (minutes)						
Per SD increment	1.23 (1.12–1.35)	<0.001	1.24 (1.12–1.36)	<0.001	1.13 (1.02–1.26)	0.019
0	1.02 (0.76–1.35)	0.916	1.00 (0.75–1.33)	0.993	1.10 (0.81–1.49)	0.555
1–30	1.00 (ref)		1.00 (ref)		1.00 (ref)	
31–60	1.33 (0.98–1.79)	0.065	1.32 (0.98–1.78)	0.071	1.23 (0.89–1.69)	0.205
61–90	1.23 (0.81–1.86)	0.340	1.23 (0.81–1.88)	0.326	1.06 (0.68–1.66)	0.801
>90	2.00 (1.39–2.87)	<0.001	1.97 (1.37–2.84)	<0.001	1.65 (1.12–2.43)	0.012
Nighttime sleep duration (hours)						
Per SD increment	0.97 (0.88–1.06)	0.463	0.96 (0.87–1.05)	0.387	1.01 (0.91–1.11)	0.922
<5	2.07 (1.22–3.51)	0.007	2.18 (1.28–3.71)	0.004	2.16 (1.23–3.79)	0.007
5-6	1.18 (0.92–1.51)	0.183	1.19 (0.93–1.53)	0.163	1.09 (0.83–1.42)	0.539
>6-7	1.00 (ref)		1.00 (ref)		1.00 (ref)	
>7-8	1.09 (0.85–1.40)	0.493	1.10 (0.86–1.41)	0.455	1.13 (0.87–1.48)	0.360
>8	1.48 (1.03–2.12)	0.033	1.47 (1.02–2.12)	0.036	1.50 (1.02–2.20)	0.038

OR, odds ratio; CI, confidence interval; ref, reference.

Model 1: unadjusted any covariate; Model 2: adjusted for age, sex; Model 3: adjusted for age, sex, educational attainment, residence, BMI, daytime nap duration (continuous), nighttime sleep duration (continuous), elevated HbA1c, hypertension, smoking, drinking, regular exercise, duration of diabetes and family history of dyslipidemia. Each group adjusted for the other covariates except itself.

### 3.7 Sensitivity analyses

To evaluate the robustness of our findings, three sensitivity analyses were conducted. First, additional adjustment for lipid-lowering therapy, glucose-lowering therapy, insulin use and antihypertensive medication was performed beyond the covariates included in the fully adjusted model (Model 3). As shown in Supplementary Table S1, the associations remained largely consistent with the primary results, with daytime nap duration >90 min significantly associated with higher odds of dyslipidemia (OR = 1.68, 95% CI: 1.13–2.51), and both short (<5 h: OR = 1.83, 95% CI: 1.01–3.35) and long nighttime sleep (>8 h: OR = 1.56, 95% CI: 1.03–2.36) remaining significantly associated with increased odds. Second, to assess the impact of the reference category definition for daytime nap duration, participants with no nap (0 min) were combined with those with very short naps (1–30 min) into a single reference group (0–30 min). As presented in Supplementary Table S2, the association between nap duration >90 min and dyslipidemia remained significant (OR = 1.56, 95% CI: 1.13–2.15), with effect sizes comparable to the primary analysis, while no significant associations were observed for naps of 31–60 or 61–90 min (both *p* > 0.05). Third, given the high prevalence of dyslipidemia (>10%), modified Poisson regression model was used to estimate PRs. As shown in Supplementary Table S3, the PRs were slightly attenuated compared with the ORs but maintained the same direction and statistical significance: nap duration >90 min (PR = 1.18, 95% CI: 1.02–1.37), short sleep <5 h (PR = 1.24, 95% CI: 1.04–1.46), and long sleep >8 h (PR = 1.17, 95% CI: 1.01–1.36). Overall, these sensitivity analyses support the robustness of the primary findings.

### 3.8 Exploratory analyses of individual lipid abnormalities

In exploratory analyses using the same covariate adjustment as in Model 3, we examined the associations of daytime nap duration and nighttime sleep duration with four individual lipid abnormalities (Supplementary Table S4). Compared with the reference group (1–30 min), daytime nap duration >90 min was significantly associated with hypercholesterolemia (OR = 3.31, 95% CI: 1.67–6.54), hypertriglyceridemia (OR = 1.49, 95% CI: 1.01–2.21), and low HDL-C (OR = 1.58, 95% CI: 1.05–2.39). For nighttime sleep duration, compared with >6–7 h, short sleep (<5 h) was significantly associated with hypertriglyceridemia (OR = 1.83, 95% CI: 1.07–3.14) and low HDL-C (OR = 1.45, 95% CI: 1.09–1.93). No other daytime nap or nighttime sleep duration categories showed statistically significant associations with any of the four lipid abnormalities.

## 4 Discussion

The observed prevalence of dyslipidemia was 53.69% in this study. Although this estimate is slightly below the 59.3% reported in a prior study of diabetic patients by Yaru Li et al. (28), it nonetheless far exceeds the 34.0% prevalence documented in the broader Chinese general population (29). Within the context of the substantial diabetes caseload in Zhejiang Province (30), the elevated dyslipidemia burden observed here underscores the importance of identifying modifiable behavioral factors, including sleep patterns, that may contribute to lipid abnormalities in this high-risk population.

The present study yielded three principal findings regarding the relationship between sleep behaviors and dyslipidemia among T2DM patients in Eastern China. First, prolonged daytime napping was associated with higher prevalence and odds of dyslipidemia, exhibiting a graded dose–response relationship. Second, nighttime sleep duration demonstrated a clear non-linear, U-shaped association with dyslipidemia; after multivariable adjustment, both short and long sleep durations correlated with significantly elevated odds, with the lowest odds observed at approximately 7 h per night. Third, these sleep behaviors differentially impacted specific lipid components: prolonged napping (>90 min) was linked to hypercholesterolemia, hypertriglyceridemia, and low HDL-C, whereas short nighttime sleep (<5 h) was primarily associated with hypertriglyceridemia and low HDL-C. Notably, neither behavior was significantly associated with elevated low-density lipoprotein cholesterol (LDL-C). Importantly, these primary associations remained robust in sensitivity analyses: additional adjustment for lipid-lowering therapy, glucose-lowering therapy, insulin use, and antihypertensive medications yielded consistent effect sizes; redefining the reference category for daytime nap duration (0–30 min) did not materially alter the associations; and modified Poisson regression, used to account for the high prevalence of dyslipidemia, produced PRs that were slightly attenuated but maintained the same direction and statistical significance. Overall, these findings indicate that both daytime and nighttime sleep behaviors are related to lipid metabolic disorders in T2DM, but with distinct dose–response patterns, and that the observed associations are robust to multiple analytical approaches.

The observed positive association between prolonged daytime napping and dyslipidemia may reflect several interrelated biological pathways. Excessive napping is often considered a marker of disrupted circadian regulation and reduced sleep efficiency at night, which can lead to misalignment of central and peripheral clocks (31, 32). Such circadian disruption has been linked to impaired lipid metabolism through altered expression of clock genes regulating hepatic lipid synthesis and clearance (33–35), providing a plausible mechanistic pathway for the hypercholesterolemia observed among prolonged nappers in our study. In addition, longer daytime naps may be associated with reduced physical activity, which can decrease skeletal muscle lipid oxidation and promote triglyceride accumulation, a process that strongly aligns with the hypertriglyceridemia and low HDL-C observed among prolonged nappers in our exploratory analyses (36, 37). Another possible mechanism involves neuroendocrine dysregulation, including altered sympathetic nervous system activity and hypothalamic–pituitary–adrenal axis function, which may contribute to unfavorable lipid profiles through increased free fatty acid mobilization and hepatic lipid synthesis (38, 39). It is also plausible that prolonged napping reflects underlying fatigue, systemic inflammation, or subclinical cardiometabolic dysfunction, all of which are known to be associated with dyslipidemia in T2DM populations (40, 41). Notably, the association of prolonged daytime napping was observed across multiple lipid abnormalities, including elevated TC, elevated TG, and reduced HDL-C. This pattern may indicate that excessive daytime napping is linked to broader disturbances in lipid regulation rather than isolated abnormalities in a single lipid fraction. The particularly strong association with hypercholesterolemia may reflect altered hepatic cholesterol homeostasis secondary to circadian disruption, although the underlying mechanisms remain uncertain and warrant further investigation.

The U-shaped association between nighttime sleep duration and dyslipidemia suggests that both insufficient and excessive sleep may adversely affect lipid homeostasis through distinct physiological mechanisms (4, 42, 43). Short sleep duration may activate sympathetic overactivity and increase cortisol secretion, leading to insulin resistance and enhanced hepatic very-low-density lipoprotein (VLDL) production. Given that VLDL is the primary transporter of triglycerides, this mechanism provides a physiological basis for our finding that short sleep primarily drives hypertriglyceridemia and reduced HDL-C (44–46). Sleep restriction is also associated with increased inflammatory activity, including elevated interleukin−6 and C-reactive protein levels, which can interfere with lipid metabolism and promote atherogenic dyslipidemia (47, 48). In contrast, prolonged sleep duration may be a marker of poor sleep quality, underlying chronic illness burden, or low-grade inflammation (49). Extended sleep has also been linked to reduced energy expenditure and physical inactivity, which may impair lipid clearance and promote adiposity-related lipid abnormalities (50, 51). Furthermore, abnormal sleep duration in either direction may disrupt circadian rhythmicity, thereby affecting the temporal coordination of lipid synthesis and catabolism (52–54).

When compared with existing literature, our findings are broadly consistent with prior epidemiological studies reporting associations between sleep behaviors and cardiometabolic risk profiles. For instance, evidence from the Dongfeng–Tongji Cohort Study among middle-aged and older Chinese adults showed that, independent of other coronary heart disease (CHD) risk factors, individuals with nighttime sleep duration ≥10 h or daytime nap >90 min had increased risks of CHD incidence, accompanied by unfavorable changes in lipid profiles compared with those reporting 7–<8 h of sleep or 1–30 min of napping (55). Additional studies further support the link between sleep behaviors and metabolic dysregulation. Short nighttime sleep (<5 h) has been associated with poorer glycemic control in patients with T2DM (56), while longitudinal evidence from the China Health and Retirement Longitudinal Study indicates that persistent short sleep duration is related to a higher risk of subsequent multimorbidity (57). Moreover, prolonged daytime napping has been associated with reduced insulin sensitivity (58), which may contribute to adverse lipid metabolism. However, findings across studies are not entirely consistent. One study reported that long sleep duration may be associated with a lower risk of cardiovascular disease (59), whereas another study showed that both short and long sleep durations are associated with increased risks of cardiovascular events and mortality among individuals with T2DM (60). These discrepancies may be explained by differences in study populations, categorization thresholds for sleep duration, and covariate adjustment strategies. In addition, residual confounding from unmeasured factors, such as sleep quality, sleep disorders, or depression, cannot be excluded. In this context, our study extends existing evidence by simultaneously examining daytime napping and nighttime sleep duration within a single T2DM population and by identifying distinct dose–response patterns, including a linear relationship for daytime nap duration and a U-shaped relationship for nighttime sleep duration. This dual-pattern finding suggests that different dimensions of sleep behavior may have independent and non-equivalent metabolic implications. Moreover, the exploratory analyses of individual lipid abnormalities suggest that these associations may be driven predominantly by disturbances in TG and HDL-C metabolism, findings that are biologically plausible given the close links between sleep regulation, insulin resistance, and triglyceride-rich lipoprotein metabolism.

From a clinical perspective, these findings suggest that both excessive daytime nap and abnormal nighttime sleep duration are associated with lipid abnormalities in patients with T2DM and may represent potential targets for behavioral modification. Future prospective studies are needed to confirm these associations and to clarify whether modifying sleep behaviors may contribute to lipid health.

However, several limitations should be acknowledged. First, the cross-sectional design precludes causal inference, and reverse causation cannot be excluded; for example, dyslipidemia or related comorbidities may influence sleep duration or daytime napping patterns. Second, although a multistage random sampling strategy was used, sampling weights and cluster-robust standard errors were not applied. The potential impact of the sampling design on variance estimation and statistical inference therefore cannot be completely excluded. In addition, 171 participants were excluded due to missing data. Comparisons between included and excluded participants could not be performed, and consequently, the possibility of selection bias cannot be ruled out. Third, both daytime nap and nighttime sleep durations were assessed using single-item, self-reported questions rather than validated questionnaires or objective measures (e.g., actigraphy or polysomnography), which may introduce recall bias and non-differential misclassification. Similarly, self-reported history of dyslipidemia could be subject to information bias, although laboratory data were used to improve outcome classification. Fourth, although major clinical covariates were adjusted, residual confounding from unmeasured factors remains possible. These include obstructive sleep apnea, insomnia, depression, and other unmeasured factors. Furthermore, detailed information on specific lipid-lowering agents, dosages, and treatment duration was unavailable, and this lack of granularity may introduce residual confounding into our exploratory analyses. For instance, the absence of a significant association between sleep behaviors and LDL-C could have been partially masked by targeted statin therapy, which specifically lowers this lipid fraction. Finally, the study population was drawn from a single region in Eastern China, which may limit generalizability.

## 5 Conclusion

In conclusion, among patients with T2DM, longer daytime nap duration was associated with higher odds of dyslipidemia in a linear dose–response pattern, whereas nighttime sleep duration showed a U-shaped association, with both short and long sleep durations associated with higher odds of dyslipidemia. Exploratory analyses further suggested that prolonged daytime napping was associated with hypercholesterolemia, hypertriglyceridemia, and low HDL-C, whereas short nighttime sleep was associated primarily with hypertriglyceridemia and low HDL-C. Given the cross-sectional nature of the study, causal relationships cannot be inferred. Prospective studies are warranted to confirm these associations and to clarify the potential mechanisms linking sleep behaviors and dyslipidemia in this population.

## Data Availability

The raw data supporting the conclusions of this article will be made available by the authors, without undue reservation.
