# Supplementary material for: Daytime nap and nighttime sleep duration in relation to dyslipidemia among type 2 diabetes patients in eastern China: a cross-sectional study
Source: Front Nutr. 2026 Jun 30;13:1861408. doi: 10.3389/fnut.2026.1861408 (PMC13364963; doi:10.3389/fnut.2026.1861408)
Supplement: Supplementary file 1 [file Supplementary_file_1.DOC]

**Supplementary:**

**Table S1. Association of daytime nap and nighttime sleep duration with dyslipidemia after additional adjustment for glucose-lowering therapy, insulin use and anti-hypertensive medication (n=1,749)**

| Characteristics | OR(95%CI) | *p* |
| --- | --- | --- |
| Daytime nap duration (minutes) |  |  |
| 0 | 1.09(0.80-1.50) | 0.582 |
| 1-30 | 1.00(ref) |  |
| 31-60 | 1.27(0.91-1.76) | 0.163 |
| 61-90 | 1.05(0.66-1.67) | 0.843 |
| >90 | 1.68(1.13-2.51) | 0.011 |
| Nighttime sleep duration (hours) |  |  |
| <5 | 1.83(1.01-3.35) | 0.046 |
| 5-6 | 1.09(0.82-1.45) | 0.533 |
| >6-7 | 1.00(ref) |  |
| >7-8 | 1.16(0.87-1.54) | 0.305 |
| >8 | 1.56(1.03-2.36) | 0.037 |

Abbreviations:OR, odds ratio; CI, confidence interval; ref, reference.

Adjusted for age, sex, educational attainment, residence, BMI, daytime nap duration (continuous), nighttime sleep duration (continuous), elevated HbA1c, hypertension, smoking, drinking, regular exercise, duration of diabetes and family history of dyslipidemia, antihypertensive medication, lipid-lowering therapy, glucose-lowering therapy, and insulin use. Each group adjusted for the other covariates except itself.

**Table S2. Association of daytime nap duration with dyslipidemia using combined reference category (*n*=1,749)**

| Characteristics | OR(95%CI) | *p* |
| --- | --- | --- |
| Daytime nap duration (minutes) |  |  |
| 0-30 | 1.00(ref) |  |
| 31-60 | 1.14(0.89-1.44) | 0.296 |
| 61-90 | 0.98(0.66-1.46) | 0.937 |
| >90 | 1.56(1.13-2.15) | 0.007 |

Abbreviations:OR, odds ratio; CI, confidence interval; ref, reference.

Adjusted for age, sex, educational attainment, residence, BMI, daytime nap duration (continuous), nighttime sleep duration (continuous), elevated HbA1c, hypertension, smoking, drinking, regular exercise, duration of diabetes and family history of dyslipidemia. Each group adjusted for the other covariates except itself.

**Table S3. Sensitivity analysis using modified Poisson regression model for the association of daytime nap and nighttime sleep duration with dyslipidemia (*n*=1,749)**

| Characteristics | PR(95%CI) | *p* |
| --- | --- | --- |
| Daytime nap duration (minutes) |  |  |
| 0 | 1.02(0.89-1.17) | 0.737 |
| 1-30 | 1.00(ref) |  |
| 31-60 | 1.08(0.95-1.24) | 0.249 |
| 61-90 | 1.01(0.83-1.22) | 0.956 |
| >90 | 1.18(1.02-1.37) | 0.027 |
| Nighttime sleep duration (hours) |  |  |
| <5 | 1.24(1.04-1.46) | 0.014 |
| 5-6 | 1.00(0.90-1.12) | 0.945 |
| >6-7 | 1.00(ref) |  |
| >7-8 | 1.05(0.94-1.18) | 0.383 |
| >8 | 1.17(1.01-1.36) | 0.039 |

Abbreviations: PR, prevalence ratio; CI, confidence interval; ref, reference.

Adjusted for age, sex, educational attainment, residence, BMI, daytime nap duration (continuous), nighttime sleep duration (continuous), elevated HbA1c, hypertension, smoking, drinking, regular exercise, duration of diabetes and family history of dyslipidemia. Each group adjusted for the other covariates except itself.

**Table S4. Association of daytime nap and nighttime sleep duration with individual lipid abnormalities (n=1,749)**

| Characteristics | Hypercholesterolemia | | Hypertriglyceridemia | | High LDL-C | | Low HDL-C | |
| --- | --- | --- | --- | --- | --- | --- | --- | --- |
|  | OR(95%CI) | *p* | OR(95%CI) | *p* | OR(95%CI) | *p* | OR(95%CI) | *p* |
| Daytime nap duration (minutes) |  |  |  |  |  |  |  |  |
| 0 | 1.20(0.62-2.34) | 0.582 | 0.87(0.62-1.21) | 0.406 | 0.73(0.40-1.35) | 0.319 | 1.25(0.89-1.77) | 0.200 |
| 1-30 | 1.00(ref) |  | 1.00(ref) |  | 1.00(ref) |  | 1.00(ref) |  |
| 31-60 | 1.43(0.73-2.82) | 0.302 | 1.22(0.87-1.73) | 0.255 | 1.04(0.56-1.91) | 0.909 | 1.40(0.98-2.01) | 0.063 |
| 61-90 | 1.53(0.63-3.76) | 0.348 | 1.25(0.77-2.02) | 0.362 | 1.01(0.44-2.36) | 0.975 | 1.44(0.89-2.35) | 0.139 |
| >90 | 3.31(1.67-6.54) | <0.001 | 1.49(1.01-2.21) | 0.046 | 1.14(0.57-2.29) | 0.710 | 1.58(1.05-2.39) | 0.028 |
| Nighttime sleep duration (hours) |  |  |  |  |  |  |  |  |
| <5 | 0.70(0.23-2.08) | 0.516 | 1.83(1.07-3.14) | 0.028 | 1.07(0.40-2.89) | 0.889 | 1.45(1.09-1.93) | 0.011 |
| 5-6 | 0.89(0.55-1.45) | 0.640 | 1.02(0.77-1.36) | 0.880 | 0.90(0.54-1.50) | 0.685 | 1.59(0.91-2.79) | 0.106 |
| >6-7 | 1.00(ref) |  | 1.00(ref) |  | 1.00(ref) |  | 1.00(ref) |  |
| >7-8 | 0.82(0.50-1.36) | 0.452 | 0.96(0.72-1.29) | 0.805 | 0.88(0.52-1.48) | 0.623 | 1.16(0.87-1.56) | 0.313 |
| >8 | 0.84(0.41-1.72) | 0.627 | 1.08(0.72-1.62) | 0.711 | 0.63(0.27-1.48) | 0.292 | 1.41(0.94-2.10) | 0.093 |

Abbreviations: LDL-C, low density lipoprotein-cholesterol; HDL-C, high density lipoprotein-cholesterol; OR, odds ratio; CI, confidence interval; ref, reference.

Adjusted for age, sex, educational attainment, residence, BMI, daytime nap duration (continuous), nighttime sleep duration (continuous), elevated HbA1c, hypertension, smoking, drinking, regular exercise, duration of diabetes and family history of dyslipidemia. Each group adjusted for the other covariates except itself.
